# Mental health problems of children and adolescents, with and without migration background, living in Vienna, Austria

**DOI:** 10.1186/s13034-019-0295-y

**Published:** 2019-09-10

**Authors:** Maria Teresa Gutmann, Metin Aysel, Zeliha Özlü-Erkilic, Christian Popow, Türkan Akkaya-Kalayci

**Affiliations:** 1 Department of Child and Adolescent Psychiatry and Psychotherapy, General Hospital Baden-Mödling, Fürstenweg 8, 2371 Hinterbrühl, Austria; 2 Department of Child and Adolescent Psychiatry and Psychotherapy, University of Zürich, Rämistrasse 71, 8006 Zurich, Switzerland; 3 Outpatient Clinic of Transcultural Psychiatry and Migration-Induced Disorders in Childhood and Adolescence, Department of Child and Adolescent Psychiatry, Medical University of Vienna, Währinger Gürtel 18-20, 1090 Vienna, Austria; 4 Department of Child and Adolescent Psychiatry, Medical University of Vienna, Währinger Gürtel 18-20, 1090 Vienna, Austria

**Keywords:** Mental health, Psychological disorders, Turkish-speaking migrants, Children, Adolescents, Migration background

## Abstract

**Background:**

Compared to their indigenous peers, migrant children and adolescents are at increased risk for mental health problems. The aim of our study was to compare psychological disorders of children and adolescents with Turkish migration background and their native Austrian peers.

**Methods:**

We analysed 302 children and adolescents aged between 7 and 18 years. The sample consisted of 100 Austrian and 100 Turkish outpatients with mental health problems, and 102 healthy controls, 52 with Austrian and 50 with Turkish background, recruited from various Viennese local child and youth centres.

**Results:**

Native patients had more frequently externalizing problems (42.1%) compared to the Turkish-speaking sample (28%). However, in the control group, Turkish-speaking children and adolescents had higher levels of internalizing, depressive and anxiety symptoms compared to their native peers.

**Conclusions:**

We found noticeable differences in psychological problems among children and adolescents with and without migration background. We assume that migration-related stress factors are responsible for these differences. Also, children and adolescents with migration background seek for psychological help less frequently than their indigenous peers.

## Background

Worldwide, approximately 20% of children and adolescents are affected by mental health problems [1] with an increasing number of chronic mental health problems [2]. Stressful life events (death of a closely related person, parental divorce, serious illness etc.) chronic stress, e.g. due to school problems, conflicts with family members or peers, may be in the background of these mental health problems [3].

Gao et al. [5] observed that mental health problems increased bimodally with age: with a minor peak between ages 6 and 10 years, and a major peak between ages 13 and 16 years. These age-related frequencies are not directly related to specific mental disorders [4]. Internalizing and externalizing problems as well as behavioural problems are more frequently observed in adolescents than in younger children [5, 6]. Major depression, e.g. is more frequently observed in adolescents than in children [7]. More boys than girls suffer from externalizing problems in their childhood [8], whereas more girls than boys undergo mental health problems during adolescence [4, 7, 9].

Boys generally are more likely to present behavioural [10] and externalizing problems [5, 11, 12], whereas, internalizing problems is more commonly observed in girls [12]. Girls usually present higher levels of anxiety [13–15], phobias [16] and depression. In addition, anxiety disorders are highly comorbid with depressive disorders [17].

A large number of studies confirm migration as a high-risk factor for mental health problems [18–20]. Migration-related stress, economic disadvantages, and discrimination increase the vulnerability of the persons concerned [21–24].

Turkish-speaking migrants living in Austria have been reported to suffer more likely from poverty and mental disorders compared to the indigenous population [25, 26] and to more frequently present symptoms such as anxiety, nervousness, discomfort and severe fatigue [27].

Migrants and especially females with migration background face more challenges and are therefore particularly more vulnerable to mental health problems [28, 29]. In addition to being more vulnerable to loss of family members, migrant children and adolescents are more exposed to acts of discrimination, racism and xenophobia [25, 30–35]. Consequently the physical [36] and mental health [34, 37, 38] of migrant children and adolescents are more endangered compared to their native peers.

The study of Diler et al. reported higher levels of depression and anxiety among migrant children and adolescents compared to their indigenous peers [39, 40]. Migrant adolescents score higher on the CBCL scales [41]: withdrawn, anxiety/depression, social problems, attention deficit, and internalizing problems [6]. Children having a low socioeconomic background face more mental health and internalizing problems [10] because they are exposed to high levels of social stress [4, 16, 42].

Shoshani et al. [43] showed that especially older and female adolescents with migration background present more mental health problems than their native peers.

Brettschneider et al. [44] found that adolescents with migration background and especially female adolescents with Turkish migration background had more severe mental health problems than their native German peers.

About 10.4% of the European population are migrants [45]. The fertility rate among migrants living in European countries is quite high; consequently, the number of children and adolescents with migration background is steadily growing [46, 47]. Regardless of the fact that 18.9% of the Austrian population had a migration background in 2012 [48] research focusing on mental health problems of migrants is scarce [49].

Providing a specialized outpatient service for migrant children and adolescents with mental health problems, we prospectively investigated children and adolescents with psychosocial problems and with or without Turkish migration background, and a group of healthy control children. The aim of the study was to analyse the transcultural background of mental health problems.

We hypothesized that mental health problems would be higher in children and adolescents with Turkish migration background and that these children would have more internalizing, externalizing, and behavioural problems, as well as increased levels of depression and anxiety.

## Methods

We prospectively investigated 200 children and adolescents aged between 7 and 18 years attending our outpatient service because of mental health problems. In the clinical sample 100 native and 100 Turkish-speaking patients were involved. A control sample of healthy, similar-aged children consisted of 50 Turkish and 52 Austrian children. They were recruited from various Austrian and Turkish child and youth centres in Vienna.

The gender distribution was quite similar in Turkish speaking and Austrian participants (F (1, 294) = 0.04, p = 0.84) and study groups (F (1, 294) = 0.09, p = 0.76). The gender distribution was different in the whole study sample (F (1, 294) = 4.34, p = 0.04), female patients were older (M = 12.11, SD = 0.26) than the males (M = 11.33, SD = 0.26).

We used six standard questionnaires for assessing psychopathology:

1. *YSR/11*-*18* (Youth Self Report for minors aged between 11 and 18 years) [50], in order to assess the internalization and externalization problems.
2. *CBCL/4*-*18* (Child Behavior Checklist for minors aged between 4 and 18 years) [51], in order to assess the internalization and externalization problems.
  Additionally Turkish version of CBCL was used [52].
3. *SES* (Self-Esteem Scale) [53], in order to assess the self-esteem.
4. *DIKJ* (Inventory for Depression of children and adolescents) [54], in order to assess depression.
5. *STAI*-*K* (Inventory for anxiety of children and adolescents aged between 7 and 14 years) [55], in order to assess anxiety.
6. *STAI* (Inventory for anxiety of adolescents aged between 15 and 18 years) [56], in order to assess anxiety.

The Ethics Committee of the Medical University of Vienna approved this study. We obtained informed consent from all children and adolescents and from their parents before including them in the study.

### Statistical analysis

For the present study, the statistical analysis was conducted with IBM SPSS Statistics 21.0. We calculated descriptive parameters, and used ANOVA and MANOVA for analysing differences in parametric data. The “Chi-Quadrat-Tests” and “Fisher´s exact tests” were used for non-parametric data, assuming significant differences at an alpha level ≤ 0.05.

## Results

### Study sample

302 children participated in the study: 152 were native Austrians, and 150 were children and adolescents with a Turkish migration background. The mean age of the whole study sample was 11.7 ± 3.1 (SD) years. The clinical group consisted of 200 patients, the control group of 102 subjects. These groups were divided according to their age in two groups, 7–11 years and 12–18 years (Table 1).

**Table 1:** Study sample

	Austrian participants		Turkish speaking participants		Total
	Female	Male	Female	Male	
Clinical group					
7–11 years	25	25	22	25	97
12–18 years	25	25	28	25	103
Control group					
7–11 years	13	14	14	13	54
12–18 years	12	13	12	11	48
Total	75	77	76	74	302

### Psychiatric diagnosis according to the ICD-10 classification

Table 2 lists the clinical diagnoses of the clinical groups and their sex distribution.

**Table 2:** Frequencies and percentages of psychiatric diagnosis depending on nationality and gender

	Nationality		Sex	
	Austrian participants	Turkish speaking participants	Female	Male
F3 (mood (affective) disorder	7 (4.6%)	9 (6%)	11 (7.3%)	5 (3.3%)
F4 (neurotic, stress-related and somatoform disorders)	18 (11.8%)	27 (18%)	27 (17.9%)	18 (11.9%)
F5 (behavioural syndromes associated with physiological disturbances and physical factors)	2 (1.3%)	1 (0.7%)	2 (1.3%)	1 (0.7%)
F6 (disorders of adult personality and behaviour)	9 (5.9%)	11 (7.3%)	15 (9.9%)	5 (3.3%)
F8 (disorders of psychological development)	0 (0%)	10 (6.7%)	6 (4%)	4 (2.6%)
F9 (behavioural and emotional disorders with onset usually occurring in childhood and adolescence)	64 (42.1%)	42 (28%)	39 (25.8%)	67 (44.4%)

Analysing differences in the distribution of diagnoses among children and adolescents with and without migration background, we observed significant differences for gender (Fisher’s Exact Test = 17.20, p = 0.01) and migration background (Fisher’s Exact Test = 18.38, p < 0.01). The clinical and control groups differed particularly in their distribution of the F9 diagnoses. F9 diagnoses were more frequently observed in males (44.4%) than in females (25.8%), and more frequently in Austrian children (42.1%) than in the Turkish-speaking sample (28%). F8 diagnoses were observed only in the Turkish-speaking sample. Age dependency was observed only as a tendency (Fisher’s Exact Test = 11.84, p = 0.06; Table 2).

### Internalizing and externalizing problems as assessed by the parents (CBCL/4-18)

#### Internalizing problems

The mean internalizing problems scale score was by 12.97 (SD = 9.61).

Significantly more (F (1, 286) = 7.36, p = 0.01) children and adolescents with Turkish migration background scored higher on the internalizing problems scale (M = 12.66) than the Austrian children (M = 9.94).

Comparing the clinical and control groups, we found significant differences between both these groups but not between the two clinical groups (Table 3). Children of the control group with a Turkish migration background (M = 8.71) compared to Austrian control children (M = 3.48) showed higher mean scores.

**Table 3:** Internalizing problems (mean ± SD) of Austrian and Turkish-speaking children

Internalizing problems	Nationality	
	Austrian participants	Turkish speaking participants
Study group		
Clinical group	16.40 SD = 0.82	16.61 SD = 0.82
Control group	3.48 SD = 1.14	8.71 SD = 1.17

We found no significant gender differences for internalizing problems between the two study groups.

### Externalizing problems

The mean externalizing problems scale score was 13.95 (SD = 12.47).

Significantly more (F (1, 286) = 11.14, p < 0.01) native patients scored higher on the externalizing problems scale (M = 20.92) than the Turkish-speaking patients (M = 15.53).

In the clinical group, the Austrian patients (M = 20.92) scored higher on the externalizing problems than the Turkish patients (M = 7.11) and the control groups did. Turkish-speaking patients (M = 15.53) had significantly more externalizing problems than native (M = 4.01) and Turkish speaking (M = 7.11) children and adolescents of the control groups (Table 4).

**Table 4:** Externalizing problems (mean ± SD) of Austrian and Turkish-speaking children

Externalizing problems	Nationality	
	Austrian participants	Turkish-speaking participants
Study group		
Clinical group	20.92 SD = 1.04	15.53 SD = 1.05
Control group	4.01 SD = 1.45	7.11 SD = 1.48

We found significant gender differences for externalizing problems (F (1, 146) = 4.84, p = 0.03). Significantly more males (M = 14.88) than females (M = 12.00) had externalizing problems.

### Internalizing and externalizing problems as assessed by the children themselves (YSR)

The mean score of internalizing problems was 15.34 (SD = 9.45) and the mean score of externalizing problems was 14.33 (SD = 8.05). The multivariate analysis showed significant differences for gender (F (2, 145) = 3.22, p = 0.04) and study group (F (2, 145) = 9.44, p < 0.01).

The influence of the migration background was not significant (F (2, 145) = 2.62, p = 0.08). The univariate analysis showed that males reported significantly more externalizing problems than females (M = 14.88 vs. M = 12.00, F (1, 146) = 4.84, p = 0.03).

The univariate analysis showed significant effects for both scales of internalizing (F (1, 146) = 7.18, p = 0.01) and externalizing problems (F (1, 146) = 16.89, p < 0.01). The clinical sample reported significantly more internalizing (M = 16.67) and externalizing (M = 16.13) problems than the controls (internalizing problems: M = 12.41, F = 7.18, p = 0.01; externalizing problems: M = 10.74, F = 16.89. p < 0.01).

### Behavioural problems as assessed by parents

The clinical group presented significantly (F (1, 286) = 157.77, p < 0.01) more behavioural problems (M = 55.33) than the control group (M = 18.37).

The multivariate analysis showed significant differences for behavioural problems between nationality and study group (F (1, 286) = 11.33, p < 0.01; Turkey’s HSD = 10.11).

The univariate analysis showed that, native children and adolescents (M = 11.70) had significantly lower scores in behavioural problems than their Turkish-speaking (M = 25.03) peers as well as Austrian (M = 58.57) and Turkish-speaking (M = 52.09) patients (Table 5).

**Table 5:** CBCL/4-18 behavioural problems (mean ± SD) of Austrian and Turkish-speaking children

Behavioural problems	Nationality	
	Austrian participants	Turkish-speaking participants
Study group		
Clinical group	58.57 SD = 2.41	52.09 SD = 2.42
Control group	11.70 SD = 3.35	25.03 SD = 3.43

Furthermore, Austrian and Turkish-speaking minors in the clinical groups showed significantly higher scores for behavioural problems compared to the control groups.

We found no significant gender differences for behavioural problems between the two study groups.

### Depression

We found significant differences for depression between the two study groups (F (1, 286) = 30.61, p < 0.01) and between the two age groups (F (1, 286) = 5.24, p = 0.02).

The clinical sample (M = 14.36) had higher depression scores than the control sample (M = 9.38). Furthermore, depression was significantly more frequent among older children and adolescents (M = 12.89) in comparison to the younger (M = 10.84) sample.

We observed significant interactions between depression score and migration background (F (1, 286) = 4.01, p = 0.05; Turkey’s HSD = 3.08).

Austrian (M = 14.61) and Turkish-speaking (M = 14.10) clinical samples had higher mean depression scores than both control groups. Whereas children of the control group with a Turkish migration background (M = 10.93) compared to Austrian control children (M = 7.83) exhibited significantly higher mean depression scores (Table 6).

**Table 6:** Depression (mean ± SD) of Austrian and Turkish-speaking children

Depression	Nationality	
	Austrian participants	Turkish-speaking participants
Study group		
Clinical group	14.61 SD = 0.74	14.10 SD = 0.74
Control group	7.83 SD = 1.02	10.93 SD = 1.05

We found no significant gender differences for depression between the two study groups.

### Anxiety

The multivariate analysis showed significant differences for anxiety between nationality (F (2, 285) = 3.63, p = 0.03), the study groups (F (2, 285) = 5.64, p < 0.01) and age groups (F (2, 285) = 5.82, p < 0.01). The group with a Turkish migration background (M = 51.21) presented higher state-anxiety scores than the Austrian group (M = 48.13).

The univariate analysis showed that Austrian subjects (M = 44.40), females (M = 45.95) and the younger group (M = 44.02) of the control sample had lower state-anxiety scores in comparison to the other groups (Table 7).

**Table 7:** Anxiety (mean ± SD) of the nationality, gender and age groups

State-anxiety	Nationality		Gender		Age	
	Austrian participants	Turkish- speaking participants	Female	Male	Age between 7 and 11	Age between 12 and 18
Study group						
Clinical group	51.86 SD = 0.93	50.55 SD = 0.93	51.59 SD = 0.93	50.81 SD = 0.93	51.35 SD = 0.94	51.06 SD = 0.91
Control group	44.40 SD = 1.28	51.59 SD = 1.31	45.95 SD = 1.29	50.04 SD = 1.30	44.02 SD = 1.26	51.97 SD = 1.34

## Discussion

Our results showed noticeable differences for mental health problems among children and adolescents with and without migration background.

### Internalizing, externalizing, and behavioural problems

Both, parental CBCL and self-assessed YSR scores were higher for internalizing and externalizing problems compared to the respective control groups.

Janssen et al. [6] reported higher behavioural problem scores for internalizing problems among youths with Turkish migration background when compared to Dutch peers. Our results support these findings. We speculate that Turkish-speaking minors are more exposed to external problems like migration-related stress, and maybe do not seek adequate professional help in time. In addition, migrant children tend to hide their problems in order not to appear feeble.

Austrian psychiatric patients exhibited higher scores for externalizing problems than their Turkish-speaking peers. Gao et al. [5] found in a Chinese sample, that children with migration background had more internalizing and externalizing mental health problems compared to their native peers. This difference may probably be explained by population-based differences in education standards and the related social behaviour.

Darwish Murad et al. [57] reported results that did not differ from our analyses. Turkish speaking adolescents reported more problem behaviour such as internalising problems than their Dutch peers. This ethnic difference between adolescents with and without migration background, may also be due to divergences in their socio-economic status [57].

### ICD-10 psychiatric diagnoses

The psychiatric diagnoses F8 (disorders of psychological development) were present only among Turkish-speaking patients. Whereas, the psychiatric disorder, F9 (behavioural and emotional disorders with onset usually occurring in childhood and adolescence) were diagnosed more frequently among native patients compared to the Turkish-speaking sample.

Akkaya-Kalayci et al., Ceri et al. and Webb et al. [24, 25, 31] also reported that specific psychiatric diagnoses were more commonly observed among migrants compared to the indigenous population. The difference may also be explained by population-based differences. On the other hand, ADHD may have a civilizing background.

### Depression

In accord with previous studies [54, 58], children and adolescents in our study had higher depression scores compared with children and adolescents of the control sample. Similar to studies [7, 9, 16, 59], we found no gender or age-related differences in our study sample but an age-dependent increase of depression symptoms.

Children of the Austrian control sample exhibited lower depression scores when compared to the Turkish control subjects and the Austrian and Turkish patients.

In line with our results, higher prevalences of depression [6, 60] and anxiety [31, 61, 62] have been reported among migrant populations. The higher depression scores may be interpreted as migration-stress related even in the 2nd and 3rd generations (LIT).

### Anxiety

Anxiety scores were elevated in both clinical groups, with no age or gender-related differences. Age and gender-related differences were only seen in the control groups. Moreover, Austrian and Turkish-speaking control groups differed in their anxiety levels. Turkish-speaking children and adolescents in our study and control groups had similar anxiety levels (Table 7). These results are partly in line with the study of Brunner [58].

Anxiety levels usually increase with age, particularly during adolescence [16, 17, 63, 64]. We only found such differences in the control groups, where females and younger children exhibited lower anxiety scores. Usually, anxiety scores are lower in boys [13, 14, 63, 65].

Migrant children and adolescents, similar to migrant adults, were described to have higher overall anxiety scores compared to autochthonous children [39, 66–68]. The study of Borgna and Contini [69] report, that children and adolescents with migration background are disadvantaged in the school system, as most of the features of the educational system do not cover the needs of migrant children. The differences in our study may be related to the specific situation of the Turkish-speaking children in the school system.

Our findings show that non-clinical Turkish-speaking children and adolescents had higher levels of internalizing, depressive and anxiety symptoms, which probably are in relation to migration-induced acculturation stress that impaired their mental health. Since the migrant population does not adequately and timely use mental health services, they only ask for professional help when severe problems occur [70].

These results can also be explained with cross-cultural differences among native Austrians and Turkish speaking migrants, especially in the cultural dimensions of individualism and collectivism. Austrians are individualistically oriented, in contrary the Turkish community is collectively oriented which makes their lifestyles quite different in many aspects [71]. Among individualistically oriented societies, members see themselves as autonomous agents, therefore their own preferences and goals have priority. In contrary, in collectivist societies, a strong connection among members of the social group is present and loyalty is quite important, so that they choose goals, which do not threaten group harmony [72]. Turkish families living in Austria are mostly very traditionally and hierarchically oriented. As the majority of the Turkish population is collectively oriented and also belongs to Islamic faith, Turkish speaking children probably suffer from acculturation stress in Austria because of the larger cultural and religious differences between the culture of origin and the dominant host culture [25, 73–75].

Furthermore, in the present study we analysed factors like socio-economic status or acculturation stress, which may be related to transcultural differences. Our results showed that children and adolescents with Turkish migration background had a lower socio-economic status compared to their indigenous peers; additionally they were impaired due to a high level of acculturative stress.

## Conclusions

Chronic mental health problems of children and adolescents increase steadily and exert a growing burden on the health-care system that suffers from increasing utilization. Therefore, preventive measures, aimed at reducing mental health problems become more and more important [76].

Migration-related illness promoting factors like acculturation stress, discrimination, racist and xenophobic politics, and economic disadvantage especially affect vulnerable children [30, 31, 35, 77–83].

Migrants, due to many reasons, use mental health services less frequently than the indigenous population although migrant children and adolescents being particularly vulnerable to mental health problems, exhibit specific needs [84, 85]. Therefore, in order to adequately treat migrant children and adolescents, mental-health services for this population should be offered culture and language sensitive care [81, 86].

Our findings are in line with previous studies, which report that migrant children and adolescents are a group that is vulnerable to mental health problems, who have special needs, therefore diversity-care measures are of utmost of importance. In Austria, language and culture-sensitive diversity-care offers are available, but what the health-care system offers is not sufficient for the entire migrant population. Due to this fact, professionals working in the health-care system should be trained to gain transcultural competence for treating migrants adequately.

At present, anti-immigrant politics prevail in Europe and the US, increasing the psychological burden especially on children and adolescents [87]. It is therefore of utmost importance to reduce racist and xenophobic propaganda, highlighting the fruitfulness of a multicultural society [88].

## Limitations

Our study bears some limitations that should be acknowledged:

1. In the control sample, Turkish-speaking families, who noticed abnormalities in their children, participated in the study, as they used the examination as an opportunity to clarify the psychological state of their children. This may be a reason why the Turkish control sample presents itself as more burdened. But this fact also means that the mental-health care of migrant children is undersupplied.
2. The study was conducted in the City of Vienna General Hospital; therefore, the results may not be generalizable to other regions of Austria or Europe.

## Data Availability

All data and material are available at the Department of Child and Adolescent Psychiatry at the Medical University Vienna.
